# A systematic review of just-in-time adaptive interventions (JITAIs) to promote physical activity

**DOI:** 10.1186/s12966-019-0792-7

**Published:** 2019-04-03

**Authors:** Wendy Hardeman, Julie Houghton, Kathleen Lane, Andy Jones, Felix Naughton

**Affiliations:** 1 School of Health Sciences, University of East Anglia, Norwich Research Park, Norwich, NR4 7TJ UK; 2 Norwich Medical School, University of East Anglia, Norwich Research Park, Norwich, NR4 7TJ UK

**Keywords:** Physical activity, Sedentary behaviour, Mobile applications, Mobile Health, Just-in-time Adaptive Intervention, Digital intervention, Telemedicine, Exercise

## Abstract

**Background:**

Progress in mobile health (mHealth) technology has enabled the design of just-in-time adaptive interventions (JITAIs). We define JITAIs as having three features: behavioural support that directly corresponds to a need in real-time; content or timing of support is adapted or tailored according to input collected by the system since support was initiated; support is system-triggered. We conducted a systematic review of JITAIs for physical activity to identify their features, feasibility, acceptability and effectiveness.

**Methods:**

We searched Scopus, Medline, Embase, PsycINFO, Web of Science, DBLP, ACM Digital Library, Cochrane Central Register of Controlled Trials, ClinicalTrials.gov and the ISRCTN register using terms related to physical activity, mHealth interventions and JITAIs. We included primary studies of any design reporting data about JITAIs, irrespective of population, age and setting. Outcomes included physical activity, engagement, uptake, feasibility and acceptability. Paper screening and data extraction were independently validated. Synthesis was narrative. We used the mHealth Evidence Reporting and Assessment checklist to assess quality of intervention descriptions.

**Results:**

We screened 2200 titles, 840 abstracts, 169 full-text papers, and included 19 papers reporting 14 unique JITAIs, including six randomised studies*.* Five JITAIs targeted both physical activity and sedentary behaviour, five sedentary behaviour only, and four physical activity only. JITAIs prompted breaks following sedentary periods and/or suggested physical activities during opportunistic moments, typically over three to four weeks. Feasibility challenges related to the technology, sensor reliability and timeliness of just-in-time messages. Overall, participants found JITAIs acceptable. We found mixed evidence for intervention effects on behaviour, but no study was sufficiently powered to detect any effects. Common behaviour change techniques were goal setting (behaviour), prompts/cues, feedback on behaviour and action planning. Five studies reported a theory-base. We found lack of evidence about cost-effectiveness, uptake, reach, impact on health inequalities, and sustained engagement.

**Conclusions:**

Research into JITAIs to increase physical activity and reduce sedentary behaviour is in its early stages. Consistent use and a shared definition of the term ‘JITAI’ will aid evidence synthesis. We recommend robust evaluation of theory and evidence-based JITAIs in representative populations. Decision makers and health professionals need to be cautious in signposting patients to JITAIs until such evidence is available, although they are unlikely to cause health-related harm.

**Reference:**

PROSPERO 2017 CRD42017070849.

**Electronic supplementary material:**

The online version of this article (10.1186/s12966-019-0792-7) contains supplementary material, which is available to authorized users.

## Background

Mobile health (mHealth) interventions have great potential to improve access to and use of behaviour change support, in addition to or instead of support delivered face-to-face, by phone, print or websites. mHealth is defined as medical and public health practice supported by mobile devices, such as mobile phones, patient monitoring devices, personal digital assistants (PDAs), and other wireless devices [1]. mHealth is especially promising for changing physical activity, defined as “any bodily movement produced by skeletal muscles that require energy expenditure” [2], and sedentary behaviour. Three meta-analyses have compared mHealth interventions aimed at promoting physical activity with usual care and support their potential for effectiveness. A meta-analysis of 21 randomised controlled trials (RCTs) found that mHealth interventions achieved a significant decrease in sedentary behaviour and a non-significant increase in total physical activity, moderate to vigorous intensity physical activity and walking compared to usual care [3]. A second meta-analysis included 15 RCTs evaluating computer, mobile and wearable technology tools to reduce sedentary behaviour. These interventions significantly reduced sitting time up to 6 months after the intervention, but only in studies with shorter follow-ups [4]. A third meta-analysis of eight RCTs of mobile phone, self-monitoring and website interventions aimed at increasing physical activity found that effects on physical activity were similar to face-to-face interventions or written materials without technology [5].

The use of mHealth technology has been recommended as portable devices make intervention delivery more interactive and responsive [3, 6, 7], for instance, by sending feedback messages in real time based on the user’s location. Progress in mHealth technology has enabled the design of interventions which aim to deliver behaviour change support in real time that is matched to when users most want or need the support. Multiple terms have been used for such interventions: just-in-time adaptive interventions (JITAIs), context-aware interventions, ecological momentary interventions (EMI) and real-time interventions. In this paper, we use the term JITAI. JITAIs have the potential to address situations when people are likely to engage in an unhealthy behaviour, for example at specific locations like a bar where people may experience an urge to smoke. They may also facilitate opportunities to perform healthy behaviours such as walking instead of taking the bus whilst commuting to work [8].

Nahum-Shani et al. [8] describe JITAIs as being designed to dynamically address the need of the user via the provision of the right amount or type of support needed at the right time. Examples described include interventions that prompt users to complete an assessment at specific times during the day, immediately followed by provision of behavioural support, such as self-management strategies if the user reports difficulties. Naughton [9] provides a definition of JITAIs as a hybrid of user-triggered and server-triggered support. Typical systems use sensors on, or connected to, the user’s smartphone (apps or other sources of data input such as online shopping habits). These are employed in real-time to interpret the context of the user’s environment and/or current mood and emotions, and deliver behaviour change support when it is appropriate, triggered by the system and not the user.

We adopt the Naughton [9] definition and define *behavioural JITAIs* as having three key features: 1) The intervention aims to provide behavioural support that directly corresponds to a need in real time when the user is at risk of engaging in a negative health behaviour or has an opportunity to engage in a positive behaviour in line with their health goal(s); 2) the content or timing of behavioural support is adapted or tailored according to input (data) collected by the system since the support was initiated, also referred to as ‘dynamic tailoring’ [10] (e.g. an app which includes the use of location sensors such as a Global Positioning System [GPS] and senses that the user is near a green space where they can be active and their digital diary shows they are not busy); and 3) behavioural support is triggered by the system and not directly by users themselves (e.g. an unsolicited mobile app notification is sent in specific situations, for instance when the in-built accelerometer has sensed a prolonged period of sitting, as opposed to users opening the app). JITAIs differ from ‘just-in-time’ interventions and tailored support because *‘just-in-time’ interventions* include features 1 and 2 but not 3; or features 1 and 3 but not 2. Likewise, *tailored support* interventions include features 2 and 3, but not 1.

JITAIs have targeted, among others, eating disorders, mental illness, obesity and weight management, physical activity [8] and smoking cessation [9], and are receiving increasing attention in the mHealth literature. JITAIs for physical activity are promising as they offer a different type of support to most existing interventions by their potential to intervene in response to changing contexts. Physical activity is strongly influenced by context such as the built environment [11], which could be exploited by JITAIs. There is a need to synthesise what is known about JITAIs for physical activity to inform whether policy makers and healthcare providers should promote JITAIs for use in health and social care settings, and to identify evidence gaps to be addressed by research. We identified two papers about the conceptualisation of JITAIs. Nahum-Shani et al. [8] report two examples of JITAIs aimed at promoting physical activity or reducing sedentary behaviour, and Muller et al. [12] identified three JITAIs aimed at increasing physical activity or reducing sedentary behaviour in older adults. However, neither conducted a systematic search for JITAIs and synthesised the evidence about the JITAIs around their effectiveness, feasibility or acceptability. A recent systematic review focused on just-in-time feedback in diet and physical activity interventions, rather than JITAIs [13]. We are not aware of any systematic reviews of JITAIs which specifically target physical activity.

The primary question for our systematic review was: (1) What are the features, feasibility, acceptability and effectiveness of JITAIs to promote physical activity? Secondary questions were: (2) Which delivery platforms have been used?; (3) Which groups or populations have been targeted?; (4) What are the active ingredients (behaviour change techniques) and theory base of JITAIs?; (5) What is the uptake and reach of JITAIs, including any impact on health inequalities?; (6) What is the level of initial and sustained engagement with JITAIs, and how have people used them?; (7) Which research designs have been used to develop and evaluate JITAIs, and (8) Has any health economic evaluation or modelling been conducted?

## Methods

### Design

This systematic review adheres to the Preferred Reporting Items for Systematic Reviews and Meta-Analyses (PRISMA) checklist. The protocol is published on PROSPERO (CRD42017070849) [14].

### Inclusion criteria

We included studies of any design, including qualitative and quantitative studies reporting data or findings from development work, feasibility or pilot studies and definitive evaluation studies. We excluded conceptual papers that did not report any data such as papers about the theory base of JITAIs or purely methodological papers. Systematic reviews and meta-analyses of mHealth interventions were excluded, but they were checked for any eligible papers about JITAIs. We included studies which aimed to promote physical activity (by increasing activity or reducing sedentary behaviour) in any setting and targeted participants of any age. Studies were included when they reported a JITAI which included the three features defined in the introduction and were excluded if they were simply ‘just-in-time’ or ‘tailored support’ interventions. Given the diverse terminology used for JITAIs, we did not rely on the use of this term during screening and decided on eligibility based on the intervention description. We included JITAIs which targeted multiple behaviours provided that a substantial part of the intervention targeted physical activity and we could extract data about the physical activity component. To be included, studies had to investigate or report one or more of the following measures: physical activity, engagement, uptake, reach, retention, feasibility and acceptability. Hence, we included studies that did not directly seek to promote physical activity among their participants providing they investigated these variables using a JITAI.

### Search strategy

We searched Scopus, Medline, Embase, PsycINFO, Web of Science, DBLP, ACM Digital Library, the Cochrane Central Register of Controlled Trials, ClinicalTrials.gov, and the ISRCTN register. Initial searches were conducted during May–June 2017 and updated searches in November 2018. They were adapted for each database and no language or date restrictions were employed. We combined search terms related to physical activity, mHealth interventions and JITAI features (Additional file 1).

### Paper screening and data extraction

All papers identified through the searches were downloaded into EndNote and duplicates removed. Screening was undertaken in EndNote in three phases. In the initial search, titles of all papers were doubly screened (JH and KL) and papers which obviously failed to meet inclusion criteria were excluded. WH resolved any discrepancies. Abstracts were screened (JH and WH) and reasons for exclusion recorded. Full texts of all remaining papers were screened for inclusion (JH, KL and WH). In the updated search, JH conducted title screening, JH and WH abstract screening, and WH full-text screening. Inter-rater agreement during the initial search was calculated by the number of papers on which two reviewers agreed (either inclusion or exclusion), divided by the total number of double-screened papers. Following the PRISMA statement [15], we recorded reasons for exclusion at the full-text screening phase.

### Data extraction and synthesis

The first author extracted data from all included papers. This was independently validated by four reviewers (FN, AJ, KL and JH) and FN independently validated the extraction of behaviour change techniques (BCTs) [16]. We extracted general information about the included papers, features of the JITAI, data about the feasibility, acceptability and effectiveness of JITAIs, delivery platforms, target groups/populations and user engagement. Data extracted about JITAI features included types of triggers (data that cause the system to respond); data used for the triggers and their source (e.g., location, GPS); technology platform; key intervention features; behaviour change techniques and theory base. Data extracted about intervention delivery included context or setting of delivery; other intervention components delivered alongside the JITAI; and any implementation issues and challenges. Finally, we extracted data about the following outcomes: feasibility, acceptability, engagement [17], effectiveness and health-economic outcomes. Given the wide range of study designs included, data synthesis was narrative.

### Assessment of study reporting

Given the diversity of study designs and the absence of an agreed tool to assess methodological quality of JITAIs, we did not formally assess risk of bias but provide a narrative summary of the key risks of bias, such as study designs used, uptake and reach. We used the mHealth evidence reporting and assessment checklist (mERA), which includes criteria for reporting what an mHealth intervention is (content), where it is being implemented (context), and how it was implemented (technical features) [18]. Each item was scored as ‘fully reported’, ‘partially reported’ (only some evidence reported) or ‘not reported’, rather than ‘yes’ or ‘no’ as many papers only provided partial evidence on the items.

## Results

### Study selection

**Fig. 1:**
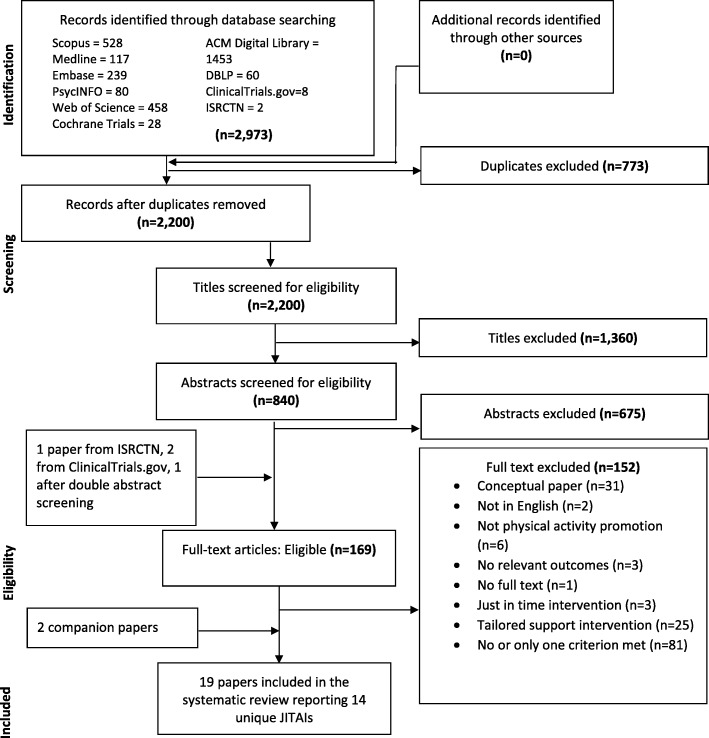
PRISMA flow diagram of study selection

In total we screened 2200 titles (Fig. 1) and checked 365/1416 titles (initial search). During abstract screening we excluded 675 out of 840 papers; key reasons were that papers were conceptual, interventions were not JITAIs or did not target physical activity. Inter-rater agreement (initial search) during double screening of 20% of abstracts (*n* = 126) was 91% (115/126). We screened 169 full-text papers: 165 from abstract screening, plus four papers following discussion of discrepancies after abstract screening [1], a search of the ISRCTN database [1] and an additional search for RCTs classified as ‘completed’ in ClinicalTrials.gov [2]. We identified three companion papers [19–21], one through paper screening and two through reading included papers and extracted any additional data. Inter-rater agreement during double screening of 20% of full-text papers (*n* = 26/132; initial search) was high: 100% for AJ and KL and 92% for JH and WH. The key reason for exclusion at the full-text phase was that the intervention did not include any JITAI feature, or only one. For instance, some interventions only provided real-time feedback on physical activity using text or graphics (e.g., a garden with flowers) [22, 23] or rewarded previous activity [24], and so were not directly targeting a real time opportunity to be active.

We included 19 papers referring to 14 unique JITAIs evaluated in 14 studies (referred to as ‘studies’ below). Two of the 14 JITAIs [25, 26] were optimised versions, informed by evaluations of earlier versions [27, 28].

### Study and participant characteristics

Eight studies were conducted in the US, five in The Netherlands and one in Portugal (Table 1). All except one [29] were described as feasibility or pilot studies; six studies [28–33] were randomised (including Van Dantzig 2013, study 2), two quasi-experimental [20, 34] and six studies reported data on feasibility, acceptability etc., but not intervention effects on behaviour (including Van Dantzig 2013, study 1) [26, 27, 33, 35–37]. Thirteen studies were conducted in the community, including three in universities [25, 31, 36], two in the workplace [29, 33] and one study in secondary care [20]. Sample sizes ranged from 6 [27] to 256 [35]; the latter study was an outlier as participants were recruited online (the next largest sample size was 86 [33]). Ten studies recruited convenience samples such as students and colleagues; these studies typically did not report any inclusion criteria. Two studies recruited at-risk groups (overweight) [30, 32] and a further two studies recruited individuals with COPD [20] and type 2 diabetes [34]. Participants were typically adults aged between 20 and 60 years of mixed gender. Most studies failed to report other participant characteristics; four studies reported education level (which tended to be high) and seven employment status.

**Table 1:** Study characteristics

Author, year and country of study	Study design	Population, inclusion criteria and setting	Sample size and recruitment method	Participant characteristics (age, gender, ethnicity, education, occupation, and socio-economic status)
Bond et al. (2014) Thomas & Bond (2015) US [30, 47]	Feasibility study/pilot evaluation. Randomised study, each participant was randomised each week to one of three conditions.	Overweight/obese adults. BMI equal to or over 25 kg/m^2^; 21–70 years. Community.	*N* = 30. Convenience sample recruited through adverts in local newspapers, research hospital network-affiliated internet/intranet and social media outlets (Facebook, Twitter).	M(SD) = 47.5 (13.5) years. 17% male. 67% white. 80% with some post high-school education. 60% employed full-time or part-time.
Ding et al. (2016) US [31]	Feasibility study/pilot evaluation. Randomised study.	College students. Participants who reported being in the contemplation and preparation stage. University.	*N* = 19. Flyers posted across the University campus and invitations sent through department’s mailing list.	*n* = 11 aged 18–20 years, *n* = 5 aged 21–25 years. 63% (10/16) male. Other characteristics not reported.
Finkelstein et al. (2015) Ouyang et al. (2015) US [32, 48]	Feasibility study/pilot evaluation. Randomised study (cross-over design).	Overweight, sedentary women. BMI > 30 kg/m^2^; inactive for more than three hours on an average day. Excluded if pregnant, unable to walk, medical reasons to limit activity, and poorly controlled hypertension. Community.	N = 30. Flyers and from previous focus groups in which participants provided feedback on messages and devices.	M(SD) = 52 (12) years. 0% male. 47% white. Other characteristics not reported.
Gouveia et al. (2015) Portugal [35]	Feasibility study/pilot evaluation. Quantitative study.	Anyone with access to Google Play. Community.	*N* = 256. Online: Habito was posted on Google Play and voluntarily downloaded.	Participants were from Portugal, US, UK, India and China. Other characteristics not reported.
He & Agu (2014) US [36]	Feasibility study/pilot evaluation. Qualitative study.	Psychology students of the Social Science Participant Pool of the Worcester Polytechnic Institute (WPI) and two participants from the Interaction Lab of the Department of Computer Science at WPI Not reported. University.	*N* = 8. Not reported.	Not reported.
Hermens et al. (2014) Tabak (2014), Chapter 6 The Netherlands [20, 49]	Feasibility study/pilot evaluation. Quasi-experimental study: single case experimental design.	People living with COPD who had completed a lung rehabilitation programme three months before the study. Clinical diagnosis of COPD, no infection or exacerbation in the four weeks prior to measurement, current or former smoker, age > 40 years, able to speak and read Dutch, and internet access at home. Exclusion criteria: other diseases with low survival rate, other diseases influencing bronchial symptoms and/or lung function, severe psychiatric illness, need for regular oxygen therapy, known alpha1-antitrypsine deficiency, disorders or progressive disease influencing daily activities, and impaired hand function causing inability to use the application. Secondary care.	*N* = 10 Hermens, *N* = 8 Tabak. Recruited from a rehabilitation centre in the Netherlands.	Age range 49–64 years (61, 60, 60, 64, 59, 64, 49, 64, 63). 60% male (6/10) 5 out of 9 participants were employed. Other characteristics not reported.
Lin et al. (2011) Lin (2013), Chapter 5 The Netherlands [19, 27]	Feasibility study/pilot evaluation. Mixed methods study.	Acquaintances (e.g. colleagues, friends) of the research group of the researchers. Colleagues and friends of the researchers. Not reported. Community.	*N* = 6. Personal contact.	M = 37 years, range 24–63 years. 83% male. 83% had completed higher education. 100% were employed.
Lin (2013), Chapter 6 The Netherlands [26]	Feasibility study/pilot evaluation. Mixed methods study.	Working population. Possession of Android phone, subscription to internet, living and working in or around Eindhoven, and working (not at home) at least four days per week. Community.	*N* = 32 of which 25 finished the user test. Through social media such as LinkedIn groups, University newspaper, newsletter by email, and the Motivate public website, and personal contacts.	M = 34 years, range 21 to 54 years. 68% (17/25) male. 96% (24/25) completed higher education. 100% were employed.
Pellegrini et al. (2015) US [34]	Feasibility study/pilot evaluation. Quasi-experimental study.	People living with type 2 diabetes. Not reported. Community.	*N* = 9. Flyers posted in the Chicago land community and online postings (e.g., Craigslist).	M(SD) = 53.1 (10.7) years. 22% male. 22% white, 77% Hispanic. Other characteristics not reported.
Rabbi et al. (JIMR, 2015) US [28]	Feasibility study/pilot evaluation, within-subject. Randomised study.	Volunteers, including students and professionals. Not reported. Community.	*N* = 17. Not reported.	M(SD) = 28.3 (6.96), range 18–49 years. 53% male. 24% professionals.
Rabbi et al. (UBICOMP, 2015) US [25]	Feasibility study/pilot evaluation. Quasi-experimental study: single case experiments.	Employees of Cornell University. Proficiency in using smartphones, in the ready or action stages of the Transtheoretical Model. University.	*N* = 16. Cornell University’s Wellness Centre’s email list.	18–29 years: 25.0%. 30–39 years: 37.5%. 40–49 years: 18.7%. > 50 years: 18.7%. 44% male. Other characteristics not reported.
Rajanna et al. (2014) US [37]	Feasibility study/pilot evaluation. Qualitative study.	Students, working professionals and people who work from home (development work). Not reported. Community.	n = 4 (ethnographic study); *n* = 47 (online study); n = 4 (prototype evaluation), *n* = 2 summative evaluation. Not reported.	Ethnographic study: 2 were working professionals, 1 worked from home, 1 student; 25% male. Online survey: 23 working professionals, 23 students, 1 ‘other’. Prototype evaluation: 4 users were students, working professionals and working from home. Summative evaluation: two graduate students in their twenties. Other characteristics not reported.
Van Dantzig et al. (2013) The Netherlands [33]	Feasibility study/pilot evaluation. Qualitative study (study 1). Randomised study (study 2).	Study 1: office workers. Study 2: healthy office workers. Study 1: not reported. Study 2: sedentary job, working at a computer not used by others, able to install the software, older than 30 years, owning a smartphone with Internet connection, no known physical handicap or other condition that makes physical activity (walking) impossible, not participating in another physical activity intervention. Study 1 and 2: Worksite.	Study 1: N = 8. Study 2: *n* = 40 intervention group, *n* = 46 control group. Study 1: not reported. Study 2: recruited via an external company.	Study 1: Age not reported. 50% male. Study 2 intervention group: 44.5 (7.9); range 30–57 years. 58% male. Study 2 control group: 44.3 (8.0); range 32–63 years. 63% male. Other characteristics not reported.
Van Dantzig et al. (2018) The Netherlands [29]	Substantive evaluation. Randomised study.	Employees. Exclusion criteria: being ill, away from work for several days, significant change in lifestyle due to external or unforeseen circumstances. Worksite.	*N* = 70. Not reported.	Age range: 18–65 years. 73% male (based on *N* = 70 recruited). Other characteristics not reported.

### JITAI features, delivery and content

We discuss a few examples illustrating the distinct features of JITAIs (see Table 2 and Additional file 2: Table S1 for details). Intervention duration ranged from 1 h [37] to 3 months [20, 35], and was typically 3 to 4 weeks. Five JITAIs targeted sedentary behaviour only [30, 32–34, 37], four physical activity only [20, 26, 27, 29], and five both sedentary behaviour and physical activity [25, 28, 31, 35, 36]. Two JITAIs also targeted calorie intake [25, 28], two provided feedback on number of calories burned [32, 36], and in one JITAI participants could set goals for keeping healthy, losing weight and burning calories [36]. Other intervention components included a face-to-face meeting to explain the use of the smartphone (in some studies participants were given a smartphone) and/or the app, to give participants devices, and explain the rationale for behaviour change (used in six studies [26, 30, 31, 34, 36]), and access to a dedicated or commercial website for physical activity feedback and instructions (used in five studies [20, 25, 28, 32, 33]).

**Table 2:** Features and delivery of just-in-time adaptive interventions

Author (year)	Real-time support: when provided and how triggered	Type of data used for real-time support, software and hardware	Content of real-time support	Theory base	Intervention duration
Bond et al. (2014) [30] Thomas & Bond (2015) [47]	Depended on the condition (three in total): 1) 3-mins break after 30 continuous sedentary minutes; 2) 6-mins break after 60 continuous sedentary minutes; 3) 12-mins break after 120 continuous sedentary minutes. Objective real-time data.	Sedentary behaviour time. Participants received an Android phone (Samsung Exhibit 4G SGH-I759).	Prompt to take a break from sedentary behaviour.	Not reported.	3 weeks.
Ding et al. (2016) [31]	When opportunistic walking moments were sensed. The message included a goal (‘take another 213 steps to reach 3000 steps!’) and a complimentary message was sent when this short-term goal was achieved. Objective real-time data.	Physical activity levels, sedentary behaviour time, smartphone use. Smartphone, Pebble smartwatch (smartwatch accelerometer used to determine whether user was eating), Android.	Prompt to walk and prompt to walk more (when already walking).	Fogg Behaviour Model, Goal Setting Theory (Locke and Latham), Habit Formation Theory.	3 weeks.
Finkelstein et al. (2015) [32] Ouyang (2015) [48]	When the person walked less than 15 steps in the past hour. No messages were sent during blackout conditions: 1) self-reported preferences collected from participant at enrolment; 2) participant texted S(X) (no messages for the next X hours), 3) participant texted ‘okay’ which meant no messages were sent during the next hour. Objective real-time data.	Physical activity levels, sedentary behaviour time. Fitbit and Android smartphone were given to the participants	Prompt to take a break from sedentary position: a tailored text message that sedentary period exceeded healthy limits, suggestions from message library on ways to have short activity breaks at work or at home, depending on the time of day.	Not reported.	4 weeks.
Gouveia et al. (2015) [35]	When participants were sedentary for 45 min. *Possibly other times and locations too but authors do not report details.* Objective real-time data.	Physical activity levels, sedentary behaviour time, and location-based sensor (details not reported). Smartphone, Android OS.	Prompt to take a break from sedentary position. *Possibly other content too given 91 different textual messages*.	Possibly Self-determination Theory (mentioned in the introduction but not intervention description); and Transtheoretical Model (used stages to categorise adoption).	10 months, but data were only analysed over a 12-week period from downloading the app.
He & Agu (2014) [36]	When participant was inactive (90% of the last 30 mins), sitting or showed sedentary patterns for an extended time. Objective real-time data.	Physical activity levels, sedentary behaviour time. GPS and time. Android OS 4.0+. Google Nexus 4 smartphone for development and testing.	Suggestions for physical activities, stand up and take a walk (when sedentary for 27 min).	Not reported.	2 weeks.
Hermens et al. (2014) [49] Tabak (2014) [20]	Suitable situations for delivery of motivational coaching (predicted by analysing previous cues and learning when a patient was likely to respond well to the message by relating relevant context factors to patient compliance and content). Objective real-time data.	Physical activity levels, previous motivational cues and ‘relevant context factors’. Hermens: smartphone and activity sensor (ProMove 3D wireless activity tracker). Tabak: HTC Desire S (smartphone) and Inertia Technology B.V. (accelerometer)	Motivational message (Hermens), prompt to walk (Tabak). Messages were encouraging, discouraging or neutral, based on physical activity levels measured in real-time.	Possibly Stages of change (mentioned in introduction and discussion but not intervention description (Tabak)).	3 months. Participants were asked to use the app at least four days per week.
Lin et al. (2011) [27] Lin (2013), Chapter 5	The system queried the geo-database, electronic diary, user profile, time and weather service, and sent support when all conditions for physical activity were met. Objective real-time data.	GPS/GSM localisation, electronic diary, weather, time and participant profile. Smartphone. Android version 2.0 and above. Software was built on Ruby on Rails platform. Participants used HTC Hero and Samsung GalaxyS.	Suggestions for physical activity.	Not reported.	4 weeks.
Lin (2013), Chapter 6	See Lin (2011)	See Lin (2011)	See Lin (2011)	Not reported.	5 weeks.
Pellegrini et al. (2015) [34]	When sedentary for more than 20 min as assessed by the accelerometer, a reminder prompt was triggered encouraging the participant to stand up for at least two minutes. Objective real-time data.	Sedentary behaviour time from a wireless accelerometer. Smartphone (Android).	Prompt to take a break from sedentary position.	Not reported.	4 weeks.
Rabbi et al. (JIMR, 2015) [28]	Based on automated sensing (accelerometer and GPS) when participants were in specific locations (on the way to work) or sedentary for prolonged period. Objective real-time data.	Physical activity levels, sedentary behaviour time. GPS. Smartphone, Android.	Suggestions for physical activities.	Learning theory, Social Cognitive Theory, Fogg’s Behaviour Model.	3 weeks.
Rabbi et al. (UBICOMP, 2015) [25]	See Rabbi et al. (JIMR 2015).	See Rabbi et al. (JIMR 2015).	See Rabbi et al. (JIMR 2015).	Decision-making theory models: multi-armed bandit and pareto-frontier. Fogg’s Behaviour Model, Social Cognitive Theory.	9 weeks (delivery ranged between 7 and 9 weeks).
Rajanna et al. (2014) [37]	After a period of sedentary time. Objective real-time data.	Sedentary behaviour time, GPS, time of day, weather, and personal calendar. Smartphone, Android.	Suggestions for physical activities	Fogg Behaviour Model, Theory of Meaning Behaviour.	1 h for the summative evaluation.
Van Dantzig et al. (2013) [33]	Study 1: when participants were sedentary for 60 min they received a prompt to take a break of 5 min, with a general daily activity goal of 50 min. Study 2: whenever 30 mins of nearly uninterrupted computer activity was recorded, a short SMS containing a hyperlink was sent to the participant’s smartphone, when clicked they were shown a message persuading them to be more active. Objective real-time data.	Sedentary behaviour time. iPhone 3G (study 1); own smartphone (study 2). Activity monitor (study 2 only) and software installed on computer to measure computer activity by registering keyboard and mouse activity.	Prompt to take a break from sedentary position.	Social influence strategies defined by Cialdini.	1 day (study 1). 6 weeks (study 2).
Van Dantzig et al. (2018) The Netherlands [29]	Support was sent during actionable moments in personally relevant geofence zones (e.g., home, work, nature area) identified in real-time based on sensor data interpretation. Objective real-time data.	Physical activity levels, time, location, weather, and behavioural events (participants achieved a step target or set a new step record). Smartphone, wrist-worn activity tracker, Philips health watch, operating system not reported.	Suggestions for physical activity, feedback about number of steps in specific contexts.	Not reported.	1 week.

Real-time support was triggered when the in-built accelerometer sensed no activity for a specified time and a prompt was sent to take a break from sitting in ten JITAIs [25, 28, 30–37]. In nine JITAIs [20, 25–29, 31, 35, 36], real-time support was triggered and activities suggested when opportunistic moments for walking or other activities were sensed. These nine JITAIs used multiple sensors, mostly in-built accelerometers and GPS, but also sensors or apps which used time of day, weather and digital diaries to identify opportunistic moments for engaging in physical activity or continuing current activity. All used apps on Android, with the exception of one study which used IOS and software installed on participants’ computers [33], and a second study which used a health watch [29]. Five studies [20, 29, 31–33] used physical activity wearables, smartwatches or activity sensors (wireless activity sensor or computer software measuring keyboard and mouse activity).

Five JITAIs were based on theory [25, 28, 31, 33, 37], most commonly Fogg’s Behaviour Model [38]. Two further studies [20, 35] mentioned a theory, but did not report explicitly how theory informed the JITAI.

We coded 19 BCTs targeting physical activity in the JITAIs (Table 3). Four BCTs were included in the majority of JITAIs: goal setting (behaviour) and prompts/cues (14/14 JITAIs); feedback on behaviour (11/14) and action planning (9/14). Goals and action plans were usually provided by the system rather than prompting users to define their own. Less frequent BCTs included discrepancy between current behaviour and goal (4/13); information about antecedents, social comparison and graded tasks (3/13). The remaining BCTs were included in one or two JITAIs only.

**Table 3:** Behaviour change techniques included in intervention and control conditions

Author (year of publication)	BCTs included in the intervention (target behaviour) * possibly; ** definitely	BCTs included in the control condition (target behaviour) * possibly; ** definitely
Bond et al. (2014) [30] Thomas & Bond (2015) [47]	Face to face session: 5.1 Information about health consequences** (PA, sedentary behaviour) JITAI:1.1 Goal setting (behaviour)** (PA)1.4 Action planning** (PA) 1.6 Discrepancy between current behaviour and goal* (PA) 2.2 Feedback on behaviour** (PA) 7.1 Prompts/cues** (PA) 10.4 Social reward** (PA) Final face-to-face visit:2.2 Feedback on behaviour** (PA)	No comparison arm. The three intervention arms differed in terms of content of the action plan and when the prompt was sent, so BCTs were identical across the intervention arms.
Ding (2016) [21]	1.1 Goal setting (behaviour)* (PA) 2.2 Feedback on behaviour** (PA) 4.2 Information about antecedents ** (PA) 7.1 Prompts/cues** (PA): sensed	1.1 Goal setting (behaviour)** (PA) 2.2 Feedback on behaviour* (PA) 7.1 Prompts/cues* (PA): randomly sent
Finkelstein et al. (2015) [32] Ouyang (2015) [48]	1.1 Goal setting (behaviour)** (PA) 1.4 Action planning** (PA) 2.2 Feedback on behaviour** (PA) 4.1 Instruction on how to perform the behaviour** (PA) 7.1 Prompts/cues** (PA)	Not applicable
Gouveia et al. (2015) [35]	1.1 Goal setting (behaviour)** (PA) 1.5 Review behavioural goal(s)** (PA) 2.2 Feedback on behaviour** (PA) 4.1 Instruction on how to perform the behaviour** (PA) 4.2 Information about antecedents* (PA) 5.1 Information about health consequences** (PA) 6.2 Social comparison* (PA) 7.1 Prompts/cues** (PA) 8.2 Behaviour substitution** (PA) 8.7 Graded tasks** (PA) 14.5 Remove reward* (PA) 10.4 Social reward** (PA)	Not applicable
He and Agu (2014) [36]	1.1 Goal setting (behaviour)** (PA) 1.3 Goal setting (outcome)** (keep healthy, lose weight, burn calories) 1.4 Action planning** (PA) 1.6 Discrepancy between current behaviour and goal** (PA) 2.2 Feedback on behaviour** (PA) 2.7 Feedback on outcome(s) of behaviour** (calories) 4.2 Information about antecedents* (PA) 7.1 Prompts/cues** (PA)	Not applicable
Hermens (2014) and Tabak (2014) [20]	1.1 Goal setting (behaviour)** (PA) 1.6 Discrepancy between current behaviour and goal** (PA) 2.2 Feedback on behaviour** (PA) 7.1 Prompts/cues** (PA)	Not applicable
Lin (2011), Lin (2013), chapter 5 (combined as the same JITAI)	1.1 Goal setting (behaviour)** (PA) 1.4 Action planning** (PA) 7.1 Prompts/cues** (PA)	Not applicable
Lin (2013), chapter 6	Optimised prototype but no changes in BCTs	Not applicable
Pellegrini et al. (2015) [34]	1.1 Goal setting (behaviour)** (PA) 2.2 Feedback on behaviour** (PA) 7.1 Prompts/cues** (PA)	Not applicable
Rabbi et al. (2015) JIMR (version 1.0)	1.1 Goal setting (behaviour)** (PA) 1.4 Action planning** (PA) 2.2 Feedback on behaviour** (PA) 2.3 Self-monitoring of behaviour** (PA) 2.5 Monitoring of outcome(s) of behaviour without feedback** (calories expended) 7.1 Prompts/cues** (PA) 8.1 Behavioural practice/rehearsal* (PA) 8.3 Habit formation** (PA) 8.7 Graded tasks* (PA)	1.1 Goal setting (behaviour)** 1.4 Action planning**
Rabbi et al. (2015) [25] UBICOMP (version 2.0)	Version 2.0 included all BCTs of version 1.0 (see above). Additional BCT in version 2.0: 1.5 Review behaviour goal(s)* (PA)	Not applicable
Rajanna et al. (2014) [37]	1.1 Goal setting (behaviour)** (PA) 1.6 Discrepancy between current behaviour and goal** (PA) 6.2 Social comparison* (PA) 7.1 Prompts/cues** (PA)	Not applicable
Van Dantzig et al. (2013) [33]	1.1 Goal setting (behaviour)** (PA) 1.4 Action planning** (PA) 1.9 Commitment* (PA) 2.2 Feedback on behaviour** (PA) 5.1 Information about health consequences** (PA) 6.2 Social comparison ** (PA) 7.1 Prompts/cues** (PA) 9.1 Credible source* (PA)	2.2 Feedback on behaviour* (PA)
Van Dantzig et al. (2018) [29]	1.1 Goal setting (behaviour)** (PA) 1.4 Action planning** (PA) 2.2 Feedback on behaviour** (PA) 4.1 Instruction on how to perform the behaviour* (PA) 7.1 Prompts/cues** (PA) 8.1 Behavioural practice/rehearsal* (PA) 10.4 Social reward** (PA)	1.1 Goal setting (behaviour)** (PA) 1.4 Action planning** (PA) 2.2 Feedback on behaviour** (PA) 4.1 Instruction on how to perform the behaviour* (PA)

*Notes*: *BCT* behaviour change technique, *PA* physical activity. Numbering refers to the BCT Taxonomy v1 [16]

### Effectiveness and cost-effectiveness

Five out of six randomised studies measured physical activity objectively, with two using independent accelerometers (Table 4). All studies collected data during intervention delivery only. Four studies reported retention rates, which ranged from 85% [30] to 100% [28]. Participants were randomised to an intervention or control group in four studies [28, 29, 31, 33], one study used a randomised crossover design [32], and another randomised each participant to three intervention conditions in succession [30]. Two studies found evidence of a positive effect. In Bond et al. [30] and Thomas et al. [47], the percentage time spent in sedentary behaviour (primary outcome) decreased significantly in all three intervention conditions, compared to baseline (*p* < .005). The intervention condition in which participants were prompted to take 3-min physical activity breaks resulted in greater decreases than the condition prompting 12-min breaks (*p* = 0.04). Finkelstein et al. [32] found that the number of episodes of prolonged inactivity (> 2 h) per day was lower (*p* < .02) when participants received inactivity reminders, compared to control when they did not receive reminders. Van Dantzig et al. [33] reported mixed findings: messages which prompted participants to reduce sitting reduced their use of the computer over six weeks, compared to the control group who received nothing, but they did not increase objectively measured physical activity. Finally, three studies found no statistically significant effects on physical activity. Ding et al. [31] showed no evidence of effects on step counts over 3 weeks of an intervention which reminded participants to walk (more) when opportunistic moments were sensed, compared to a control group who received random reminders without contextual information. Rabbi et al. [28] found a non-significant trend (*p* = .055) in self-reported walking of an intervention which provided specific suggestions based on location and activity levels: intervention participants reported an increase in walking of 10 min per day over 3 weeks, whilst no change was observed in the control group who received a digital intervention with general recommendations. Van Dantzig et al. [33] found no evidence of effects on daily step count of an intervention which sent real-time support in specific contexts, compared to messages sent at fixed times.

**Table 4:** Study findings

Author (year)	Uptake	Retention	Physical activity: measure used, how measured, follow-up period	Within- or between-group differences
Bond et al. (2014) [30] Thomas & Bond (2015) [47]	Not reported.	30 completed out of 35 consented and enrolled; 2 excluded for not following protocol and three did not complete.	Change in daily time spent in sedentary behaviour (primary outcome), daily number of minutes accrued in walking breaks. Objectively through independent measurement (separate accelerometer). Study duration: 21 days, 7 days in each condition.	Thomas: Daily number of minutes accrued in walking breaks was M(SE) = 37.24 (1.85) in the 3-mins condition and M(SE) = 38.73 (1.86) in the 6-mins condition. This did not differ between-groups, but both were significantly higher than the M(SE) = 32.49 (1.93) in the 12-mins condition. Number of daily minutes decreased significantly as a function of days. Bond: Proportion of daily time spent sedentary was M(95% CI) = 66.3 (61.7–71.0) in the 3-mins condition; M(95% CI) = 66.6 (61.5–71.7) in the 6-mins condition, and M(95% CI) = 69.0 (64.7–73.2) in the 12-mins condition. There was a significant difference (*p* < .04) between the 3-mins and 12-mins conditions in terms of change since baseline. The 3-mins condition produced significantly larger increases in proportion of time performing light intensity activity compared to the 12-mins condition (*p* = .04).
Ding et al. (2016) [31]	Two participants (one intervention, one control) did not start as app not compatible with their Samsung S4 phones.	One participant did not complete due to the app consuming too much battery power.	Step counts during weeks 2–4: perceived effectiveness of the app in terms of encouraging the participant to walk more. Objectively through in-built sensor/accelerometer (smartphone or smartwatch). Exit interview after four weeks, some data were collected at 10 pm each day (e.g. whether app encouraged them to do other activities).	Step counts during weeks 2–4: M(SD) = 40,350 (12,458) in intervention; M(SD) = 45,744 (15,541) in control group. No significant difference between intervention and control group in perceived effectiveness (M(SD) = 3.11 (0.93) versus 2.29 (0.95) on a 5-point Likert scale). Significant between-group difference in self-reported effectiveness of the app to encourage them to do other physical activities (M(SD) = 3.22 (0.67) versus 1.43 (0.79); *p* = .0002).
Finkelstein et al. (2015) [32] Ouyang et al. (2015) [48]	Not reported.	27/30 enrolled completed the study.	Number of episodes of prolonged inactivity (> 2 h) per day when the inactivity reminder was active, compared to not active. Objectively through in-built sensor/accelerometer. Eight weeks, of which four weeks with the inactivity monitor active and four weeks with the monitor inactive.	Inactivity expressed as fraction of consecutive two-hour slots between 8 am and midnight during which steps are less than 20. Inactivity was significantly lower (*p* < 0.02) during “message-on” periods (24.6%) as compared to the “message-off” periods (30.4%). Group A: M(SD) = 0.32 (0.23) during message-off period and M(SD) = 0.22 (0.14) during message on period. Group B: M(SD) = 0.28 (0.12) during message-off period and M(SD) = 0.28 (0.14) during message on period. Within-group difference between the two periods was significant (*p* < .004) for group A but not significant for group B. Higher but non-significant step counts in both groups during the message-on period compared to the message-off period (within group).
Gouveia et al. (2015) [35]	Not reported.		Distance walked per day (km). Objectively through in-built sensor/accelerometer. First 12 weeks of intervention delivery (out of 10 months).	Participants who updated their goal walked more per day (median(IQR) = 6 (3–10) km) than those who did not (median(IQR) = 2 (1–4); *p* < .05. There was a positive association between goal and distance walked per day (p < .05), and a negative correlation between goal set and accomplishment (*p* < .01).
He & Agu (2014) [36]	Not reported.	One participant dropped out due to loss of interest in the study and did not care about research credits (compensation).	Not reported.	Not reported.
Hermens et al. (2014) [49] Tabak (2014) [20]		Hermens: 2/8 patients provided activity data for analysis. Tabak: One participant dropped out after four weeks due to personal reasons, one stopped after six weeks due to non-COPD related medical reasons.	Mean activity per day for those days on which at least six hours of data were available. Other measures included whether the participants reached their physical activity goal, the number of days when accumulated activity was between 90 and 110% of the goal, and the number of days on which the balance goal was reached (> 90%). Objectively through independent measurement (separate accelerometer); and 6-min walking test to measure exercise capacity. During the three months of intervention and for one week at three months after the intervention.	Data were reported for each participant. Five participants increased their activity levels and four participants improved their activity balance between baseline (one week before the start of the intervention) and the end of the three-month intervention. Three participants had a clinically significant improvement in exercise capacity of > 25 m and one patient of 24 m. At three-month follow-up, no participant had maintained their increases in activity levels and only two participants maintained their improved activity balance. The percentage of days on which goals were achieved ranged between 23 to 59% for activity levels and between 21 and 85% for balance.
Lin et al. (2011) [27] Lin (2013), Chapter 5	Not reported.	Not reported.	Not reported.	Not reported.
Lin (2013), Chapter 6	Not reported.	Not reported.	Perceived change in physical activity. Self-report. End of intervention period.	18/21 reported that they had become a little or much more active.
Pellegrini et al. (2015) [34]	Not reported.	8/9 completed the intervention and were followed up.	Proportion of the day spent sedentary and proportion of the day spent in light physical activity. Objectively through independent measurement (separate accelerometer). One month, which was during the intervention period.	Data are reported within-participants. 7/8 participants reduced the proportion of day spent sedentary and increased time spent in light physical activity: in these 7 participants, sedentary time decreased by M(SD) = 8.1 (4.5)%,* p* = .003) between baseline and one month, and light physical activity increased by M(SD) = 7.9 (5.5)%, *p* = .009. Inclusion of the eighth participant reduced the decrease in sedentary time to the level of a trend (*p* = .08) and maintained a significant increase in light activity (*p* = .047). Breaks in sedentary time decreased over the month by M(SD) = 15.8 (8.8) (*p* = .003); whereas break duration increased by 1.0 (0.5) mins (*p* = .002).
Rabbi et al. (JIMR, 2015) [28]	Not reported.	N = 17 completed the 3-week period.	Behaviour change from participants’ logs of daily activity. Self-report. Collected during the three weeks of intervention delivery.	78% of participants in intervention group showed positive trends (upward trend to longer walks from first to third week) whereas 75% of control group participants showed negative trends (*p* = .05). Intervention group walked 10 min per day more during the 3 weeks (within group) whereas control group participants showed no change (between group-difference in change in walking duration *p* = .055).
Rabbi et al. (UBICOMP, 2015) [25]	Not reported.	Not reported.	Behaviour change from participants’ logs of daily activity. Self-report. Data collected during the 2–4 weeks of control condition and subsequent 7–9 weeks of intervention condition; total study duration was 14 weeks maximum.	Significant improvements (within-participant) were found over the final three intervention weeks (compared to 2–4 weeks of control condition) in minutes of walking per day (intervention M(SD) = 24.9 (7.4); control M(SD) =14.5 (5.9); d = 1.41; *p* < .005) and calories burned through non-walking exercise per day (intervention M(SD) = 126.7 (35.3); control M(SD) = 83.5 (33.1); d = 1.23; *p* < .05). Patterns over time showed that minutes of walking per day did not change much during the intervention period, and non-walking exercises were maintained during this period.
Rajanna et al. (2014) [37]	Not reported.	Not reported.	Not reported.	Not reported.
Van Dantzig et al. (2013) [33]	Not reported.	Not reported.	Computer activity (proxy for sedentary behaviour) and physical activity during the 30 mins before a text message were compared with computer activity and physical activity during 30 mins after the text message. Objectively through independent measurement (separate accelerometer); computer activity through specially installed software. Collected during the 6-week intervention period (study 2).	Study 2: Average computer activity before the text message: 28.3 (SD 0.32) mins in the intervention and 27.7 (0.43) mins in the control group. Average computer activity after the text message: 18.3 (4.0) mins in the intervention group (10.0 mins reduction within-group) and 21.8 (2.9) mins in the control group (5.9 mins reduction). The decline in computer activity was higher in the intervention than control (*p* < .001). Physical activity (proportion of active minutes) before the text message: 0.68 (SD 0.49) in the intervention group and 0.42 (0.26) in the control group. After the text message: 0.71 (0.41) in the intervention and 0.47 (0.24) in the control group. The between-group difference was not significant. Subgroup analyses showed that reduction in computer activity did not differ as a function of the number of messages read, so the text message only was sufficient to trigger a break and persuasive content may not be needed.
Van Dantzig et al. (2018) [29]	Not reported.	Ten participants were excluded because they did not meet one ormore of the study criteria (e.g., they had been ill or away from their work for several days, or their lifestyle had varied drasticallydue to external or unforeseen circumstances).	Average daily step count per participant. Objectively through independent measurement (separate accelerometer). Collected during a 2-week calibration period, 1-week intervention period and 1-week fade-out period.	The authors do not report precise figures for intervention and control group. Mean daily step count appears to be approx. 8200 in the intervention group and 7600 in the control group during the intervention period, and 7500 in intervention and 7600 in control during the fade-out period. Between-group differences were not statistically significant. Authors divided each group into three clusters: cluster 1: steps<=6500, cluster 2: 6500 < steps<=9500, and cluster 3: steps> 9500. The increase in mean step count from calibration to coaching period for intervention and control groups: Cluster 1: 16 and 19%; Cluster 2: 17 and 6%, and Cluster 3: − 4% and − 7%, respectively. Step counts in each cluster and group were as follows. Calibration period: M(SD) in cluster 1 intervention (*n* = 10) = 5273 (594); cluster 1 control (*n* = 11) = 4866 (1125); cluster 2 intervention (*n* = 15) = 7708 (786); cluster 2 control (*n* = 17) = 7616 (854); cluster 3 intervention (*n* = 3) = 11,200 (1675); cluster 3 control (*n* = 4) = 11,383 (728). Intervention period: M(SD) in cluster 1 intervention = 6115 (985) (larger than during the calibration period, *p* = 0.037); cluster 1 control = 5771 (1496) (larger than during the calibration period (*p* = 0.042) and fade-out period, *p* = 0.01)); cluster 2 intervention = 9002 (1877) (larger than during the calibration period, *p* = 0.007, and fade-out period, *p* = 0.035); cluster 2 control = 8088 (1965) (non-significant differences with calibration and fade-out period); cluster 3 intervention = 10,789 (2118) (all differences non-significant); cluster 3 control = 10,597 (474) (all differences non-significant). Fade-out period: M(SD) in cluster 1 intervention = 5481 (732); cluster 1 control = 5903 (1095); cluster 2 intervention = 8055 (1413); cluster 2 control = 7864 (2036); cluster 3 intervention = 10,569 (1581); cluster 3 control = 11,356 (2522).

Four non-randomised studies reported physical activity data. Two used independent accelerometers and reported retention rates of 60% at 3 months [20] and 89% at one-month follow-up [34]. They found mixed evidence for an intervention effect. In Tabak [20], 5/10 participants increased objectively measured physical activity between baseline and three-month follow-up, but no participant had maintained increases at 3 months. Pellegrini et al. [34] found a trend for an objective decrease in sedentary time (*p* = .08), and significant increase in light physical activity (*p* = .047) between baseline and 1 month. Two interventions resulted in self-reported increases in physical activity within participants. Rabbi et al. [25] found increases in minutes of walking per day (*p* < .005) over the final three intervention weeks compared to control. In one study [26], 86% of participants reported at the end of the intervention that they had become a little or much more active. The remaining four studies reported no data on intervention effects on physical activity or sedentary behaviour as they focused on feasibility and acceptability [27, 35–37].

No study reported data on resource use or cost-effectiveness.

### Uptake, reach, retention, engagement, feasibility and acceptability

Only one study reported the uptake or reach of JITAIs [31]: two out of 19 participants failed to start the app due to incompatibility with their phones (Table 4). Reasons for loss to study follow-up included battery problems, loss of interest, personal and medical reasons.

A wide range of engagement measures were used in nine studies which reported data (Additional file 3: Table S2). They included number of days or weeks that users carried smartphones [30] or used the app [26, 35], adherence to and evaluative responses of real-time messages [20, 27, 28, 30, 31], and frequency, duration and nature (‘glance’, ‘engage’ and ‘review’) of usage sessions [35]. No study reported that participant engagement was a challenge, but intervention duration was generally short. One study [35] reported that all 256 participants recruited online had quit the app by ten months.

Feasibility measures varied widely. Technological challenges included short battery life of the smartphone [33, 34, 36], the GPS requiring substantial battery power [26], technology only working when smartphone and intervention accelerometers had wireless connection [34], and participants not carrying smartphones when at work and exercising [37]. Other challenges included sensing and delivery of just-in-time messages: the digital diary was unreliable during weekends as participants did not update it [27], 43% of just-in-time messages were received too early (17%) or too late (26%) (users were asked after each notification whether it was just-in-time, too early or too late) [27]. In qualitative interviews some participants found physical activity suggestions hard to follow and not reflecting their activity preferences [28].

In terms of acceptability, participants liked short-term graded goals and explanations of why reminders to walk (more) were triggered [31], activities which could be easily integrated in daily life [26, 27], and they reported high acceptance and willingness to keep using the JITAI [32]. In qualitative interviews, just-in time messages were perceived to be more timely than random messages and resulted in less complaints compared to the control group who received non-context aware, random messages. Intervention participants reported becoming more aware of opportunities to walk, whereas this was not the case for control participants [31]. Rabbi et al. [28] reported that the number of physical activity and dietary suggestions participants wanted to follow (*βi* = 2*.*9*, p <* 0*.*001) and relatedness of suggestions to life (*βi* = 0*.*5*, p <* 0*.*001) was higher for just-in-time messages compared to randomly selected messages. JITAI participants followed suggestions more than control participants even when there were barriers (*p <* 0*.*001*, d* = 0*.*44) or when they experienced negative emotions (*p <* 0*.*001*, d* = 0*.*55). In qualitative interviews, some participants reported that suggestions were actionable and relevant to their lives; participants who considered increasing their activity were eager to follow them and the messages reinforced maintenance [28]. In another study [29] intervention participants liked real-time suggestions to build activity into their daily routines, but some did not want to be disturbed during daily activities and preferred activity during dedicated times. Features that participants did not like included being told too frequently that they were inactive [36] and participants sharing their activity data with others on social media [33], though in one study participants could not share their data on social media but reported that this would be highly motivating [37].

### Quality of intervention descriptions

At least eight out of 13 studies (the two Lin et al. interventions were treated as one as the descriptions were identical) reported full details on intervention delivery (mERA item #4), intervention content (#5), formative research and user testing (#6) and user feedback (#7) (Table 5). In contrast, at least nine out of 12 studies did not report any details on integration into existing health information systems (#3), cost-assessment (#9), user information and training (#10), solutions for delivery at scale (#11), adaptation of the intervention (#12), data security/confidentiality protocols (#14) and alignment with national and regulatory guidelines (#15).

**Table 5:**
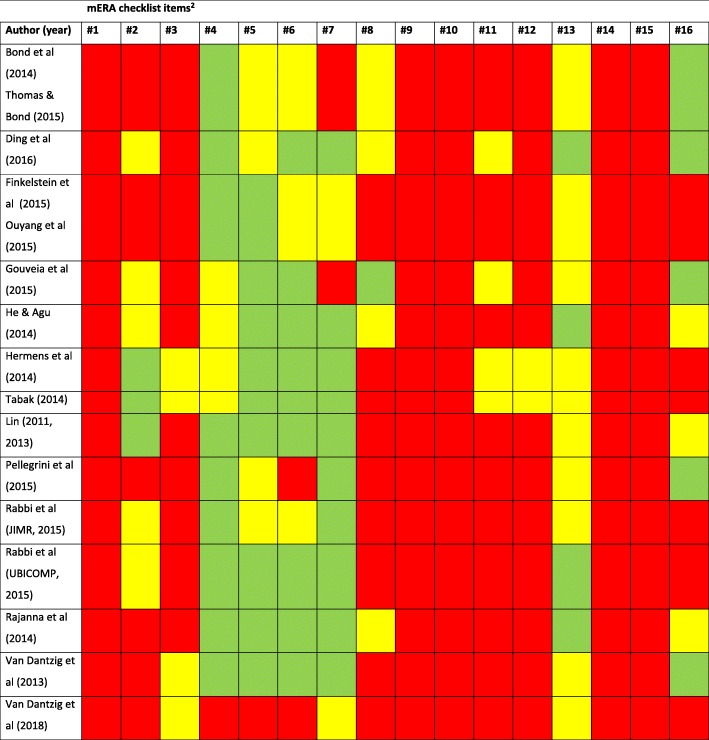
Quality of intervention descriptions, assessed with the mERA checklist [18]^1^

^1^ Green cell = fully reported. Yellow cell = partially reported. Red cell = not reported

^2^ Explanation of the mERA items (see [18] for full descriptions): 1: Clearly presents the availability of infrastructure to support technology operations in the study location. 2: Describes and provides justification for the technology architecture. 3: Describes how mHealth intervention can integrate into existing health information systems. 4: The delivery of the mHealth intervention is clearly described. 5: Details of the content of the intervention are described. Source and any modifications of the intervention content is described. 6: Describes formative research and/or content and/or usability testing with target group(s) clearly identified, as appropriate. 7: Describes user feedback about the intervention or user satisfaction with the intervention. 8: Mentions barriers or facilitators to the adoption of the intervention among study participants. 9: Presents basic costs assessment of the mHealth intervention from varying perspectives. 10: Describes how people are informed about the programme including training, if relevant. 11: Clearly presents mHealth solution limitations for delivery at scale. 12: Describes the adaptation, or not, of the solution to a different language, different population or context. 13: Detailed intervention to support replicability. 14: Describes the data security procedures/ confidentiality protocols. 15: Mechanism used to assure that content or other guidance/information provided by the intervention is in alignment with existing national/regulatory guidelines and is described. 16: Was the intervention delivered as planned?

## Discussion

We identified a heterogeneous set of 19 papers evaluating 14 unique JITAIs, mostly among convenience samples of young and middle-age adults. The JITAIs prompted breaks following sedentary periods and/or suggested physical activities during opportunistic moments. Five out of 14 JITAIs reported a theory base. Common behaviour change techniques were goal setting (behaviour), prompts/cues, action planning and feedback on behaviour. Intervention duration was typically three to four weeks. We found mixed evidence for intervention effects on physical activity and sedentary behaviour. However, no study was designed to be powered to detect effects on behaviour and only six studies were randomised. Feasibility challenges related to the technology, reliability of sensors and timeliness of just-in-time messages. There was a lack of evidence about cost-effectiveness, uptake, reach, impact on health inequalities, sustained engagement, and integration in existing systems.

Crucial evidence to inform the adoption of JITAIs in health and care systems is their added value compared to mHealth interventions without just-in-time messages (e.g., scheduled, fixed or random). Two studies supported the assumption that JITAIs deliver support when users are most likely to be receptive. Rabbi et al. found that, compared to randomly selected messages, participants intended to follow the just-in-time messages more, found them more relevant to their life, and followed them more when they experienced barriers or negative emotions [28]. In Ding et al., participants found just-in-time messages more timely than random messages, they increased their awareness of opportunities to walk, and resulted in less complaints [31]. However, this potential is only fully realised if messages are indeed just-in-time. The one study which assessed this found that 43% of messages were reported not to be just-in-time [27].

Paper screening was challenging due to a lack of shared terminology about what constitutes a JITAI and lack of clarity in descriptions of JITAI features. We used a published definition which distinguishes JITAIs from interventions which provide feedback on physical activity in real time, either numerical or through visual displays, without explicit prompts to further increase activity. We cannot assume that these interventions include ‘incentives’ or ‘rewards’ as the BCT Taxonomy v1 [16] specifies that these need to be valued by the users. A randomised controlled trial showed that provision of personalised feedback on physical activity alone does not change behaviour [39]. Our definition specifies that behaviour change support in real time needs to include additional BCTs (e.g., prompts/cues, goal setting and action planning) beyond feedback or reinforcement of previous activity to encourage further increases in activity. On this basis, we excluded some studies [22, 23, 40] which others described as JITAIs. It was also challenging to draw the line due to poorly described interventions. We urge mHealth researchers to report their intervention features clearly, whether they are JITAI or non-JITAI, and to achieve consensus about what constitutes a JITAI.

Our review advances the evidence base about JITAIs for physical activity. We chose to consider both physical activity and sedentary behaviour as they are closely aligned, recognising that others consider them as distinct behaviours. We identified 18 studies of which only three [30, 33, 36] were included in a previous review [41] which focused on design features and one [30] in a systematic review of just-in-time feedback [13]. None of our studies was included in recent meta-analyses of mHealth interventions, possibly because most studies were not RCTs and any RCTs were published recently [3–5]. Prior to this systematic review there was uncertainty about the feasibility, acceptability, effectiveness and cost-effectiveness of JITAIs for physical activity. Our review has provided initial insights on feasibility challenges, illuminated that users found JITAIs acceptable, and found mixed evidence in terms of effectiveness and lack of evidence about cost-effectiveness. However, many evidence gaps remain and our systematic review corroborates earlier reports that research into JITAIs for physical activity is in its early stages [12]. Many JITAIs appeared to be designed without behavioural science input, as they lacked a theory and evidence-base to underpin content.

We identified major evidence gaps, informing an ambitious research agenda in terms of target groups, study design and JITAI development work. In terms of target group, JITAIs need to be evaluated among representative real-world and clinical populations, including uptake, reach and impact on health inequalities, to inform decisions whether to adopt them in health and social care settings. To reach all smartphone users, JITAIs need designing for both IOS and Android smartphones and be potentially incorporated into smartwatches and other wearables.

In terms of study design, studies need to assess the additional value of just-in-time support by comparing JITAIs to interventions providing not just-in-time support, and assess whether messages were just-in-time, relevant to context, setting and motivational state, assess participants’ receptiveness to the messages and their intention to act on the messages, whether they followed any suggestions, and intervention engagement over time. Studies need to include independent objective measurement of physical activity and sedentary behaviour and follow-up beyond the end of the intervention. There is also a need for mixed methods process evaluation with a broad range of measures: engagement, fidelity, how participants use JITAIs, measures along the hypothesised mechanism of intervention effect and contextual factors. This would illuminate how, when, and for whom JITAIs work and inform adaptation and integration of JITAIs into health and care settings, a key evidence gap in the wider mHealth literature about physical activity [42].

We recommend that developers of JITAIs use appropriate theory and evidence-based BCTs [16], use design principles for JITAIs [43] and design a logic model. For instance, socio-ecological theory includes broader determinants of individual behaviour (social and physical environment) which could inform just-in-time support. The JITAIs included a small number of BCTs and lacked evidence-based BCTs targeting motivation and maintenance such as problem-solving, reviewing behavioural goals, social support, information about behavioural consequences and habit formation. Developers could consider a wider range of messages beyond prompting breaks in sitting and suggesting activities alone to encourage and maintain behaviour change and sustain engagement over time. All JITAIs used behavioural data (physical activity levels, sedentary behaviour time) or location data to trigger just-in-time support. None used data about psychological and affective states (motivation, mood, stress levels) which can influence physical activity and sedentary behaviour, perhaps due to the challenges of sensing these states. We recommend that research into JITAIs addresses these challenges and incorporates this evidence into the development of JITAIs which use data about psychological and affective states to trigger support*.* We recommend that authors use the mERA checklist to improve intervention descriptions, report BCTs included and which intervention features are JITAI and non-JITAI. JITAIs need to be developed in co-production with users and could employ innovative methodologies such as MOST [44] and SMART [45] to optimise JITAIs prior to definitive evaluation.

We recommend that decision makers adopt JITAIs for physical activity promotion when research evidence shows that they increase physical activity or reduce sedentary behaviour, and if such evidence is unavailable, that they incorporate evaluation. Although JITAIs are unlikely to cause harm if walking and sitting less is their main aim, we need evidence about their effectiveness and cost-effectiveness. Health and care professionals can signpost or refer people to evidence-based mHealth support for physical activity, including JITAIs. Industry should work with developers to optimise battery life and the delivery of just-in-time support.

The strengths of our systematic review include a comprehensive search of ten databases, a clear definition of JITAIs to guide paper screening, the identification of studies that had not been previously synthesised, and in-depth synthesis focusing on study design, intervention content and delivery, and a broad range of outcome and process measures. Our review also had limitations. The absence of an agreed definition of a JITAI made the search for papers and screening challenging, compounded by lack of clear reporting of JITAI features and diversity in reporting across disciplines (computer science, engineering, behavioural science). Consequently, we may not have identified all eligible papers, although inter-rater agreement during full-text screening was high. In addition, it was challenging to judge the quality of intervention descriptions, using the mERA checklist. Finally, we considered physical activity as any movement requiring energy expenditure, and therefore sedentary time as any time not spent in physical activity, whilst others regard physical activity and sedentary behaviour as distinct. This is an ongoing topic of research and debate [46]. We acknowledge the distinction, as well as that changing these two broad categories of behaviour may require different intervention approaches.

## Conclusions

Research into JITAIs to increase physical activity and reduce sedentary behaviour is in its early stages and consistent use and a shared definition of the term ‘JITAI’ will aid evidence synthesis. We need robust outcome and process evaluations of theory and evidence-based JITAIs in representative populations and examination of their integration in health and care systems. Decision makers and health and care professionals need to be cautious in signposting patients to JITAIs until such evidence is available, although JITAIs which target walking and sedentary behaviours are unlikely to cause health-related harm.

## Additional files

Additional file 1: Search terms used for physical activity, mHealth interventions and JITAIs. (DOCX 32 kb)

Additional file 2: **Table S1.** Description of intervention and control conditions. (DOCX 44 kb)

Additional file 3: **Table S2.** Engagement with, and feasibility and acceptability of the just-in-time adaptive interventions. (DOCX 43 kb)

## Abbreviations

BCT: Behaviour change technique
EMI: Ecological momentary interventions
GPS: Global Positioning System
JITAI: Just-in-time adaptive intervention
mERA: mHealth evidence reporting and assessment
mHealth: Mobile health
PRISMA: Preferred reporting items for systematic reviews and meta-analyses
RCT: Randomised controlled trial
