# Supplementary material for: A systematic review of just-in-time adaptive interventions (JITAIs) to promote physical activity
Source: Int J Behav Nutr Phys Act. 2019 Apr 3;16:31. doi: 10.1186/s12966-019-0792-7 (PMC6448257; doi:10.1186/s12966-019-0792-7)
Supplement: Supplementary file 1 — Search terms used for physical activity, mHealth interventions and JITAIs. (DOCX 32 kb) [file 12966_2019_792_MOESM1_ESM.docx]

**Additional file 1 – Search terms used for physical activity, mHealth interventions and JITAIs**

**Physical activity**

Physical activity, exercise, physical fitness, walk* and active living.

**mHealth interventions**

Smart phone*, “mobile phone*, text messag*, SMS, mobile PRE/2 app, mobile health, mHealth, smartphone*, cell phone*, app, application W/15 smart phone*, application W/15 mobile phone*, application W/15 mobile health, application W/15 mHealth, application W/15 smartphone*, application W/15 cell phone*, mobile technolog*, mobile application*, technology based and mobile devices. Search terms related to intervention features included: just-in-time, JITAI, ecological momentary intervention*, EMI, real time intervention*, context aware*, context triggered, context tailor*, dynamic tailor*, real time tailor*, sensor triggered, geofenc*, context sens*, real time context*, persuasive technolog*, sensing technolog*, activit* track*, fitness track*, Fitbit, Garmin, TomTom, Jawbone and Withings.

**Features of just-in-time adaptive interventions**

“Just-in-time”, JITAI, “ecological momentary intervention*”, EMI, “real time intervention*”, “context aware*”, “context triggered”, “context tailor*”, “dynamic tailor*”, “real time tailor*”, “sensor triggered”, “geofenc*”, “context sens*”, “real time context*”, “persuasive technolog*”, and “sensing technolog*”.
