# Supplementary material for: A systematic review of just-in-time adaptive interventions (JITAIs) to promote physical activity
Source: Int J Behav Nutr Phys Act. 2019 Apr 3;16:31. doi: 10.1186/s12966-019-0792-7 (PMC6448257; doi:10.1186/s12966-019-0792-7)
Supplement: Supplementary file 2 — Table S1. Description of intervention and control conditions. (DOCX 44 kb) [file 12966_2019_792_MOESM2_ESM.docx]

**Table S1 (additional file 2): Description of intervention and control conditions**

| **Author (year)** | **Description of JITAI, other intervention components delivered alongside the JITAI, and control condition** |
| --- | --- |
| Bond et al (2014)  Thomas & Bond (2015) | *JITAI*: Bond: The accelerometer monitored prolonged bouts of sedentary behaviour and prompted brief walking breaks upon reaching a clinically significant sedentary behaviour threshold. A persistent display on the screen showed minutes until the next walking prompt, total sedentary behaviour minutes, total daily physical activity minutes, and daily count of walking prompts met. Participants were asked to carry the smartphone at all times. Monitored sedentary data were available to the research team in real time and used to inform the subsequent automated goal setting, prompting and feedback. There was an audible prompt with on-screen text reminder when a break was due ('Get up and activate those muscles!'). Participants could perform the break, ignore it or delay the prompt until the next break. If the break was met then a reinforcement message and green light appeared. |
|  | *Other intervention components*: 10-mins face-to-face meeting with staff member who provided education on the rationale of reducing sedentary behaviour: definition of sedentary behaviour, risks and benefits of breaking up sedentary time. The staff members explained the smartphone and the app, and each condition participants were randomised to. |
|  | *Control condition(s)*: No comparison group. There were three intervention groups (all digital interventions), with prompts for a 3, 6 or 12-minute walk, respectively. Participants followed each condition for 7 days. |
| Ding et al (2016) | *JITAI*: WalkMore sent prompts to walk and an explanation of why the prompt was sent (context), as linking context with behaviour would facilitate habit formation. Participants were sent reminders to walk or walk more when opportunistic moments were sensed. These included: 1) participants overused their smartphone; 2) participants had been sedentary for a long time; 3) participants were walking, which presented a good opportunity to walk more; 4) participants just had their meals. |
|  | *Other intervention components*: Face-to-face session in which the app was installed, app features explained and smartwatch provided. |
|  | *Control condition(s)*: Digital intervention. Control group received motivational reminders encouraging them to walk, but these were sent randomly in time and no contextual information was considered when reminders were sent. |
| Finkelstein et al (2015)  Ouyang et al (2015) | JITAI: Tailored text message were sent after less than 15 steps were sensed in the past hour, provided that it was not a blackout period as indicated by the participant at enrolment and in real time. Participants received a daily summary of their steps during the previous day. Group A received inactivity reminders for four weeks and then nothing for the following four weeks. Group B did not receive anything for four weeks and then inactivity reminders for four weeks. |
|  | *Other intervention components*: Fitbit website where participants could see the number of steps per day, number of steps climbed, distance walked, and calories burned. |
|  | *Control condition(s)*: Nothing. |
| Gouveia et al (2015) | *JITAI*: Habito has three design features: goal setting, contextualising physical activity with cues relating to location and daily commutes, and textual feedback that kept updating with the goal of sustaining users’ interest. Goal setting: participants were prompted to define their daily walking goal, but a default goal of 1 km/day was provided. The goal was split in four sub-goals and Habito rewarded the participant when they achieved sub-goals and motivated them to achieve the next sub-goal. Contextualising physical activity: a new location was created if the participant spent at least five minutes in a 50-metre radius, and it could be associated with a name (home, work). Textual feedback was either persuasive or informative. A total of 91 messages were sent at different times and conditions, for instance, highlighting locations where participants were most active during the past week, facts about physical activity and just in time recommendations (“you've been sitting for 45 minutes, try taking a break every 30 minutes”), and creating a sense of community (“your workplace is the second most active one”). |
|  | *Other intervention components*: None. |
|  | *Control condition(s)*: Not applicable. |
| He & Agu (2014) | *JITAI*: On11 continuously monitored calories burned and percentage of the day spent sitting, walking, jogging and other activities. At-a-glance summaries were presented to participants for reflection. Based on the participant's context (location, time, personal information (e.g. electronic calendar, body weight, age), and weather) convenient walking suggestions and detours were suggested. The participant could set performance goals, which allowed the system to suggest activities which helped achieve these goals. If participants asked for a recommendation, the system predicted the next location and suggested walking routes to from the current to the predicted location. On11 has three modules: an activity logger, an inactivity detector, and a recommendation generator. The main screen showed a weather dashboard and an overview for the day of how many calories the participant had burned, how much progress they had made towards their goal, and how many minutes they had been sitting, walking, and running. Walking detour suggestions (based on GPS sensing) were displayed as different maps with time, intensity (different colours) and calories required. When sitting for extended times, the display flashed and there was a sound or vibration. Participants could input their height, weight, gender and age to get more accurate estimates of calories. They could select three types of goals: keep healthy, lose weight and burn calories. |
|  | *Other intervention components*: Face to face session to install the app. |
|  | *Control condition(s)*: Not applicable. |
| Hermens et al (2014)  Tabak (2014) | *JITAI*: The app (Activity Coach) was developed for people living with long-term conditions and evaluated among people with COPD. The smartphone received physical activity data from the sensor, provided visual continuous feedback in graphs which displayed accumulated measured activity together with the cumulative activity the patient should aim for: the reference line. The reference line was based on baseline activity levels, which were distributed over the day (40% morning, 30% afternoon, 30% evening). The reference line was increased with 10% above the mean of the measurements in the past week. Participants received text-based motivational cues which were based on the difference between measured activity and the reference line. The text message included a short summary of activity behaviour and advice on how to improve behaviour (e.g., “you took more rest, we advise you to take a short walk”). Three types of motivational texts were used: 1) encouraging (>10% deviation below reference line), 2) discouraging (>10% deviation above reference line); 3) neutral (<10% deviation with reference line). The activity coach predicted the optimal timing of the motivational cue using machine learning. Decision rules defined the content of the message and used a factor of randomness and in decision making and different representations to reduce repetitiveness of messages over time. Goals were tailored (current activity versus target) by the physiotherapist who set individual targets for patients weekly; this process was automated in later adaptations of the system. The system used seven tailoring techniques Informed by a literature review: feedback, inter-human interaction, adaptation, participant targeting, goal setting, context awareness and self-learning. |
|  | *Other intervention components*: Website where participants could log in and see their measured activity per day, week and month. |
|  | *Control condition(s)*: Not applicable. |
| Lin et al (2011)  Lin (2013), chapter 5 | *JITAI*: The system included 34 pieces of advice comprising 20 types of activities (e.g., taking the stairs, housework, gardening, walking/cycling to the park, shopping, going to a movie, walking/cycling to work). Suggested activities depended on whether the conditions for physical activity were met: the suggested activity had to be within a certain distance or travel time (location), the participant had a free slot in their app-based agenda (diary), outdoor activity required good or fair weather (weather), the participant was able to do the activity (profile), and the suggested activity was suitable for the specific time (time). Participants could see what advice they had received and the frequency of replying positively or negatively to a message. They could set preferences for the time interval when the system checked their location, the notification sound and other settings. If the system found a message a notification was sent (e.g., “it is good weather outside, why about a short walk to [*location*] during lunchtime?”). Participants could respond with “yes I will do it now”, “yes I will do it later”, “yes because I am already doing or have planned to something similar”, “no I will not do it now or later”, and “no I will never follow this kind of advice”. Participants could also type in the reason for their response. They were then asked whether the message was just in time, too early or too late. During the first week, no advice was given but data were collected on participants’ location, particularly their home and work place. |
|  | *Other intervention components*: A set-up meeting where participants downloaded the app and edited their personal profile and agenda. |
|  | *Control condition(s)*: Not applicable. |
| Lin et al (2013), chapter 6 | *JITAI*: Same as Lin (2011) plus optimised features: self-reported action where participants could report whether or not they had followed the advice; small changes in the message interface; the addition of a reminder function so that the message was sent again later (time selected by the participant); an overview of messages sent and responses by the participant; an event service through which events could be sent to all participants; and small changes in the location method and weather settings. |
|  | *Other intervention components*: Set-up meeting where instructions about downloading and using the app were given. Detailed instruction videos and descriptions were also available online. |
|  | *Control condition(s)*: Not applicable. |
| Pellegrini et al (2015) | *JITAI*: After 20 minutes of sedentary time the app initiated a noise or vibration prompt (based on the participant’s preferences) and encouraged the person to stand up. Participants were asked how they would react to the prompt: 1) stand or light ambulation for more than two minutes; 2) extend, i.e. finish the sedentary bout by 1-19 minutes; 3) cannot stand, e.g., in a work meeting; and 4) ignore. The sedentary counter was reset when sit to stand transition was detected. If the participant selected 'stand' but this was not detected, a reminder was sent every two minutes until the participant stood up or chose a different response. |
|  | *Other intervention components*: The technology was explained as part of a one-hour baseline visit; followed by a one-hour orientation session in which they downloaded the intervention (NEAT!) on their smartphone and received an intervention accelerometer (Shimmer). |
|  | *Control condition(s)*: Not applicable. |
| Rabbi et al (JIMR, 2015) | *JITAI*: Physical activity tracking: the accelerometer and GPS converted data into walking, running, stationary (sitting and standing) and driving. Participants had a list of about 800 activities to choose from and recorded start and finish time of the activity, and MyBehavior calculated calories expended. Life-log generation: a chronological list of activity events (automated and manually logged), activity predictions were made (e.g., 50 mins of stationary activity, or mixed activity such as 'taking bus from home to work'). Physical activity clustering: automatically tracked activities were clustered by the place where they occurred using machine learning. Suggestion generation: automatic generation of suggestions based on past physical activity which were performed frequently (low effort, 'exploit') and burned more calories; and sometimes infrequent high-calorie activity ('explore'); small changes were suggested for stationary activities (e.g., 3 mins break). If activities were taken up then MyBehavior learned to use this as an 'exploit' rather than 'explore' suggestion. Each day participants received ten activity suggestions, 90% were their frequent behaviours ('exploit') and 10% infrequent behaviours ('explore'). |
|  | *Other intervention components*: The JITAI also targeted calorie intake. Face-to-face training session - installation and instruction on how to enter data. |
|  | *Control condition(s)*: Digital intervention. Participants received generic prescriptive recommendations generated from a pool of 42 suggestions for healthy living, such as “walk for 30 minutes”. |
| Rabbi et al (UBICOMP, 2015) | *JITAI*: As in MyBehavior 1.0: based on automated sensing (accelerometer and GPS), when participants were in specific locations (on the way to work) or sedentary for prolonged period. |
|  | *Other intervention components*: The JITAI also targeted calorie intake. Face-to-face training session to install the app and provide instructions on how to enter data. |
|  | *Control condition(s)*: Digital intervention. Participants received eight randomly selected generic prescriptive recommendations generated from a pool of 42 suggestions for healthy living, such as “walk for 30 minutes”. |
| Rajanna et al (2014) | *JITAI*: System components included: 1) an accelerometer which sensed activity by the participant using tree-based machine learning algorithms; 2) GPS which helped the app decide whether or not to generate a notification (some zones are 'do not disturb' zones, some are 'friendly' zones (home, office) and others 'non-friendly' (e.g., cinema); participants could add these zones; 3) time of the day - for instance, the participant might want longer working spells during mornings than afternoons and can adapt this; 4) weather - to suggest the most appropriate activity; 5) personal calendar – the app did not send a notification after a sedentary period if an event was scheduled, but instead five minutes after the end of this event. Notifications were sent using haptic vibrations, with duration depending on the number of times the participant had snoozed a notification. The system architecture consisted of an activity tracking system and a ‘Step Up Life’ participant interface. The app maintained a threshold for sedentary (idle) time which varied throughout the day, based on time of day and how much activity the participant had already done. If an alert was triggered the participant chose the following options: they did the suggested activity, cancelled or snoozed. When the timer ran out the participant was alerted to stop the activity. |
|  | *Other intervention components*: None. |
|  | *Control condition(s)*: Not applicable. |
| Van Dantzig et al (2013) | *JITAI*: SitCoach aimed to increase sedentary awareness and reduce sedentary behaviour. Study 1: The number of active minutes per day was stored and shared with peer participants. SitCoach used a visual, tactile or an acoustic signal. A visual screen showed when the participant was moving, and when sedentary, the app signalled the time left until the next prompt. When the break was longer than a pre-set length (default 5 mins) the timer was reset. Study 2: All messages contained the same general advice, using four persuasion strategies: authority, commitment, consensus and scarcity. In total 32 messages were designed. Participants received a maximum of three messages per day, with at least two hours in between messages. |
|  | *Other intervention components*: Study 1: none. Study 2: participants could view their activity patterns on a website. |
|  | *Control condition(s):* Nothing. |
| Van Dantzig et al (2018) | *JITAI*: Messages were delivered as push notifications with a dedicated audio cue and also appeared as ‘cards’ in the Newsfeed tab of the coaching app. Based on collected data, the remote coaching engine selected the appropriate coaching messages from the message database, containing over 500 message templates of different categories; e.g. target-setting, time- or location-triggered suggestion, location- or activity-based feedback. The intervention distinguished different event types: (i) Time events: specific clock time (e.g., 7 am), (ii) Location events: the user enters or exits one of their personal geofence zones (e.g., home, school, work) or encounters an iBeacon, (iii) Behaviour events: the user executes certain behaviour (e.g., reaching a step target, setting a new step record). Intervention participants received context-aware messages, sent during actionable moments identified in real-time based on sensor data interpretation. These messages either provided a suggestion for a specific action to be performed in the current context (e.g., “Welcome at the train station. Walk to the far end of the platform to gain extra steps”), or feedback about the number of steps taken in a specific context (e.g., “Well done! You have taken 2300 steps at work today!”). |
|  | *Other intervention components*: Each morning, intervention participants received a suggestion for a daily step target, and each evening a message providing feedback on their performance. |
|  | *Control condition(s):* Each morning, control participants received a suggestion for a daily step target, and each evening a message providing feedback on their performance. Throughout the day, control participants received messages offering general advice how to enhance physical activity, delivered at fixed, predefined moments. |
