# Supplementary material for: A systematic review of just-in-time adaptive interventions (JITAIs) to promote physical activity
Source: Int J Behav Nutr Phys Act. 2019 Apr 3;16:31. doi: 10.1186/s12966-019-0792-7 (PMC6448257; doi:10.1186/s12966-019-0792-7)
Supplement: Supplementary file 3 — Table S2. Engagement with, and feasibility and acceptability of the just-in-time adaptive interventions. (DOCX 43 kb) [file 12966_2019_792_MOESM3_ESM.docx]

**Table S2 (additional file 3): Engagement with, and feasibility and acceptability of the just-in-time adaptive interventions**

| **Author (year)** | **Engagement, feasibility and acceptability** |
| --- | --- |
| Bond et al (2014)  Thomas & Thomas (2015) | *Engagement*: Participants carried the smartphone an average of 6.90 days out of seven days. Adherence to prompts (% of prompts resulting in a walking break) was lower in the 12-min condition (M=77%) compared to the two other conditions (M=89% for 3-mins condition; 87% for 6-mins condition). Latency between prompts and start of walking breaks was the lowest in the 3-mins condition; followed by the 6 and 12-mins conditions. Prompts were responded to within 5 minutes for 41%, 41% and 34% for 3-mins, 6-mins and 12-mins condition, respectively. |
|  | *Feasibility*: Not reported. |
|  | *Acceptability*: 57% most preferred the 6-mins condition, 33% the 3-mins condition and 10% the 12-mins condition. 47% least preferred the 3-mins condition and 53% least preferred the 12-mins condition. |
|  | *Other process measures*: M(SD) of prompts was 7.01 (.25) per day in the 3-mins condition, 4.53 (.25) in the 6-mins condition and 2.29 (.25) in the 12-mins condition. This did not increase or decrease over time. 90% of participants reported that the display and feedback increased their motivation to take breaks, and 90% that messages and feedback decreased their sedentary time. |
| Ding et al (2016) | *Engagement*: Participants were more likely to 'accept' reminders (through feedback in real time) when they were walking and reminded to walk more. Reminders to take a break were less likely to be accepted due to students being in classes (but could have ‘delayed effect’, i.e. walk when finished class). |
|  | *Feasibility*: All participants found the system easy to use; the system usability score was M(SD)=83.06 (6.71) in the intervention group and M(SD) = 76.07 (9.67) in the control group (a score higher than 68 is considered average). Participants noted reminders on the smartphone less often and found the smartwatch easier to send feedback on.  Intervention participants were more likely to report than control group participants that reminders were sent out at a good time. |
|  | *Acceptability*: Participants liked the short-term graded step goals. Intervention participants reported that the explanation of why reminders were triggered, made them more convincing. Participants in the control group complained more about the timing of the reminders (e.g., already in the gym). In both groups, some reminders were sent at the wrong time, e.g., when students were in class or taking an exam. Participants suggested adding a sync schedule to the app. Participants wanted richer historical data over and above daily steps and reminders. For instance, steps walked on a weekly basis and charts. |
|  | *Other process measures*: Intervention participants received more reminders (M(SD)=20.8 (3.34)) than the control group (M(SD)=10.9 (0.52)), but reminders did not annoy them more than the control group. Most reminders were sent when participants were sitting and walking. Intervention participants reported becoming more aware of opportunities to walk, whereas this was not the case for control participants. |
| Finkelstein et al (2015)  Ouyang et al (2015) | *Engagement*: Not reported.  *Feasibility*: Not reported.  *Acceptability*: Two focus groups showed that most participants expressed high acceptance of the app and indicated willingness to use it in the future. |
| Gouveia et al (2015) | *Engagement*: 66% used Habito longer than two days, 38% longer than one week and 14% longer than two weeks. All 256 participants had quit Habito by the end of the 10 months. Participants were divided into adopters (38% i.e. those who used Habito for more than one week) and non-adopters (62%) who quit Habito within the first week. Adopters used Habito for a median (IQR) of 11 (8-16) days, and non-adopters 2 (1-4) days. Adopters (n=97) had 2,737 individual usage sessions; median (IQR) per participant was 28 (15-45). 50% of usage sessions was less than 10 secs and 81% less than 30 secs (median (IQR) = 10 (4-24) secs). 57% of all sessions were 'glance' sessions (Habito opened and closed without additional input or actions) (median (IQR)= 5(2-11)). 22% were 'review' sessions (defined as < 60 seconds) (median (IQR) = 12(8-18), and 21% were 'engage' sessions (defined as >60 seconds) (median(IQR)= 45(29-67) seconds). 88% of all review sessions and 89% of all explore sessions involved exploring the ongoing day's contextual feedback, while exploring the textual messages occurred in 84% of review and 88% of engage sessions. Accessing textual messages was higher during engage as opposed to review sessions (p<.05) and accessing past days was higher during engage than review sessions (p<.05). Engage sessions were more frequent when goal accomplishment was low, and glance sessions more frequent when participants progressed towards their walking goals (p<.05 for both). Glance sessions increased over time (45% first week to 73% in 12th week, p<.05) and engage sessions decreased over time (28% in first week to 9% in 12th week; p<.05). 46% of all usage sessions occurred during first 25% of goal accomplishment, decreasing towards goal accomplishment (p<.05). Participants took less time to re-engage with Habito after an engage session than after a glance session (p<.01). Contextual information was accessed in 38% of all usage sessions and this decreased over time (p<.05): 43% in first week to 18% in 12th week. Interactions concerned the ongoing day (89%), the past day (7%), and earlier days (4%). In 71% these were glance sessions. In 32% of all sessions participants received novel messages that they had not seen before, which made them more likely to swipe to additional messages compared to 'old' messages (p<.05). Participants took longer to re-engage with Habito after an engage sessions (p<.05) but took less time to start walking and walked for longer distances (p<.05). |
|  | *Feasibility*: Not reported. |
|  | *Acceptability*: Not reported. |
|  | *Other process measures*: Adoption showed a significant association with stages of change (measured by email when participants downloaded the app): 56% among those in contemplation and preparation, and 20% among those in pre-contemplation, action and maintenance (p<.01). 31% of adopters changed the pre-set goal during first use. 87% of adopters received a recommendation to change their goal at least once, but only 5% did. |
| He & Agu (2014) | *Engagement*: Not reported. |
|  | *Feasibility*: Battery life was a significant concern. 5 out of 8 participants reported that activity recognition worked accurately and inactivity was detected when their phones were attached to their body movements. The smartphone of one participant did not detect activity accurately. |
|  | *Acceptability*: Six participants liked the at-a-glance presentation. They did not like to be told that they were inactive too frequently, even though they knew they were. |
| Hermens et al (2014)  Tabak (2014) | *Engagement*: Compliance to individual motivational messages was higher with the adaptive system compared to historical data (62.7% versus 56.0%). |
|  | *Feasibility*: One participant stopped wearing the activity sensor after two months due to technical problems. |
|  | *Acceptability*: Not reported. |
| Lin et al (2011) Lin (2013), chapter5 | *Engagement*: Users responded to 434/464 messages (with either “Yes-Now”, “Yes-Later” “Yes-Already”, “No” and “Never”).  *Feasibility*: Participants received on average three messages a day, in the morning (45%), afternoon (25%) and evening (30%). The messages sent mostly related to work, home and indoors, and the most frequent ones were to take a break from work (15%) and take the stairs when arriving at or leaving work (11%). Participants reported that 57% of messages were received just-in-time, 26% too early and 17% too late. Participants did not update their agenda during the weekend which made the agenda function challenging: messages were sent assuming that participants were not busy but they were. |
|  | *Acceptability*: Participant interviews: they liked daily activities which were easy to do; they forgot to update their diaries during the weekend so any advice may need to be sent out in advance. |
|  | *Other process measures*: Data collected through the app: participant rated 50.5% of messages positively (reporting that they would do the suggested activity now or later or they were already doing it), and 47% of messages negatively (reporting that they would not do it now or ever). Work messages received 75% positive responses; easy to do activities like taking a coffee break or taking stairs were easiest to do and responded to positively. Participants gave a reason for 106/215 responses: too busy (25%), have other plans (20%) and feel no need to do PA (17%). Other reasons included bad weather, too tired, not feasible, don't feel like it. |
| Lin (2013), chapter 6 | *Engagement*: Motivate was used for 45 days (range 30-70 days). Participants responded to 2,848 (83.3%) of all 3,421 messages. 57% of the messages were viewed within 30 minutes, while 31% of the messages were viewed within five minutes, and the remainder mostly within 12 hours. After participants viewed the messages, they submitted their responses for 94% within two minutes. Participants used the reminder function for 40% of messages. The “Remind later” messages were followed significantly less often (66%) than the messages where no reminder was needed (81%) (χ² = 7.224, p< 0.05). |
|  | *Feasibility*: The authors initially tried to use Google agenda but this was not feasible due to challenges with data interpretation and input needed from participants. GPS in smartphones used a lot of battery power.  3,548 messages were sent to participants (*author note: this differs from the total reported under engagement but this figure is reported in the paper*). This was an average of 3-4 per day and varied between 1.4 to 5.5 messages per day across participants. In general, the messages were distributed evenly during the day. |
|  | *Acceptability*: During interviews, more than half of the participants mentioned that their favourite messages suggested easy-to-do activities such as taking a break, taking stairs, or going to a supermarket. The messages that they thought they followed least were those needing extra effort, such as going to events or doing weekend outdoor activities. Not enough time or having other plans were the major reasons for rejecting suggestions. They liked using the reminder for small activities like taking a break or taking stairs. They wanted a greater range of suggested activities, more personalised advice and smarter advice, pictures in addition to messages, and more information (calories burned, distance to green space). |
|  | *Other process measures*: 48% of messages received a positive response (including “Yes now”, “Yes later” and “Yes already”) and 52% a negative response. For 40% of messages participants reported that they had done the suggested activities, and for 60% they reported not having followed the advice. For more than 75% of the latter the reasons were “I already have other plans”, “I don’t feel like doing it” and “I am busy and have no time”.) Utilitarian activities (taking stairs, cycling to work) received more positive responses (57%) than recreational activities (30%) (χ² =179,187, p = 0.000); and participants reported following utilitarian activities more often (48%) than recreational activities (24%) (χ² =108.578, p = 0.000). The messages of taking a break and taking stairs received the most positive responses (77% and 61%). The top nine messages (n = 1,159, 41% of all 2,848 messages) were given positive responses for more than half of the time; these messages included taking small breaks, taking the stairs, and walking to the canteen and housework.  The questionnaire findings showed no change in attitude from baseline to post-intervention. A significant change in attitude was found among people who responded positively to the app. |
| Pellegrini et al (2015) | *Engagement*: During the one-month intervention period, participants used NEAT on M(SD)=21.9 (0.8) days for 7.6 (2.5) hours per day. |
|  | *Feasibility*: 8/9 participants completed the intervention. During interviews participants mentioned the short battery life of the intervention accelerometer, and the technology only worked when the smartphone and intervention accelerometer were wirelessly connected. |
|  | *Acceptability*: 75% (strongly) agreed that NEAT was easy to use, 87.5% that it did not interfere with their social life, 100% that the accelerometer did not make them feel uncomfortable around others, 75% that the intervention accelerometer did not interfere with their job, and 62.5% that the intervention accelerometer was comfortable to wear. 75% (strongly) agreed that a 20 mins sedentary period was acceptable, and 37% liked the 'can't stand' option. 87.5% intended to use NEAT in the future. |
|  | *Other process measures*: Participants selected 'stand' 62.6% of the time. All participants (strongly) agreed that NEAT made them more aware of how much time they spent sitting and motivated them to stand up, and 87.5% that NEAT made it easier to break up their sitting time. |
| Rabbi et al. (JIMR, 2015) | *Engagement*: Not reported.  *Feasibility*: On a few occasions, the suggestions were hard to follow or did not reflect activity preferences. |
|  | *Acceptability*: Semi-structured interviews with intervention participants showed that they found the suggestions actionable and relevant to their life, whereas the control group participants appreciated the messages but struggled to incorporate them in their daily lives. Intervention participants found the explore suggestions actionable and expressed interest in acting on them: those considering making changes were eager to follow the suggestions and for those maintaining an active lifestyle the suggestions were a reinforcement. Intention to follow suggestions (3-week follow-up questionnaire) was significantly higher in the intervention than control group. |
| Rabbi et al (UBICOMP, 2015) | *Engagement*: The number of suggestions followed with no barrier was M(SD)= 3.4 (2.8) in the intervention and M(SD) = 1.3 (2.2) in the control group, d=.84, p<.001. The number of suggestions followed with a barrier was M(SD)= 1.6 (2.5) in the intervention and M(SD) = 0.6 (2.1) in the control group; d=.44, p<.001. The number of suggestions followed with a positive emotional state was M(SD)= 3.2 (2.6) in the intervention and M(SD) = 1.2 (1.9) in the control group; d=.82, p<.001. The number of suggestions followed with a negative emotional state was M(SD)= 1.9 (2.1) in the intervention and M(SD) = 0.7 (1.5) in the control group; d=.55, p<.001. |
|  | *Feasibility*: Authors report that the iterative development process took 2.5 years, during which they tested several prototypes. |
|  | *Acceptability*: Authors compared data collected daily through an in-phone survey of 2-4 weeks of the control condition against the final three weeks of the intervention condition. There were significant improvements in the number of suggestions (physical activity as well as calorie restriction) followed (intervention M(SD)=3.1 (2.7) ; control M(SD) = 1.1 (1.1), d=.76; p<.0005), the number of suggestions wanted (intervention M(SD)=4.4 (2.4) ; control M(SD) =2.1 (1.2); d=1.07; p<.0005), and how well suggestions related to the participant's life (intervention M(SD)= 4.5 (1.2); control M(SD) = 3.8 (1.1); d= .54; p<.0005). Emotional states ('rate your emotional state today'), perceived barriers to following the suggestions and their interaction with the intervention improved significantly. |
| Rajanna et al (2014) | *Engagement*: Not reported. |
|  | *Feasibility*: Participants did not carry a smartphone with them all the time and did not feel comfortable wearing the phone when exercising or leaving it on their desk whilst working. |
|  | *Acceptability*: Prototype evaluation with four participants (unclear whether these were the same participants as the ethnographic study) included the presentation of real-time scenarios. Participants wanted to be convinced about security aspects, they wanted suggestions for a wide range of activities, wanted to share statistics on social media, they liked a 'tree growing' metaphor displaying the column of physical activity taken, they wanted to tailor their preferences, did not get the concept of reward, wanted to set goals and liked integration of the calendar with the app. During the evaluation 'based on functional prototype' and a summative evaluation with two participants, participants commented mostly on technical issues. |
| Van Dantzig et al (2013) | *Engagement*: Study 2 intervention participants read a M(SD) of 46% (34.6) of messages.  *Feasibility*: Study 1: Some participants reported battery problems, so the phone was not used as an assessment tool in study 2. Not all participants carried the smartphone with them all day. |
|  | *Acceptability*: Study 1: Participants reported that the time between tasks and reduced concentration were good moments to take a sitting break. The auditory (buzzing) signal was experienced to be distracting: ‘‘When I am working, I don’t want to be disturbed’’. Participants were positive about their interaction with the app but did not find it very appealing. Participants did not want to share data on social media such as Facebook. |
|  | *Other process measures*: Intervention participants in Study 2 received a M(SD) of 43 (18.5) messages. |
| Van Dantzig et al (2018) | *Engagement*: Not reported.  *Feasibility*: Not reported. |
|  | *Acceptability*: The intervention group was more positive than the control group about the coaching they had received, but there were mixed responses. Some participants appreciated the actionable suggestions to incorporate more activity into their daily routines, whereas others did not want to be disturbed during their daily duties (work, grocery shopping) and preferred to be physically active during dedicated moments throughout the week. |
|  | *Other process measures*: Not reported. |
